# Assessment of gene-covariate interactions by incorporating covariates into association mapping

**DOI:** 10.1186/1753-6561-3-s7-s85

**Published:** 2009-12-15

**Authors:** Yen-Feng Chiu, Hui-Yi Kao, Yi-Shin Chen, Fang-Chi Hsu, Hsin-Chou Yang

**Affiliations:** 1 Division of Biostatistics and Bioinformatics, Institute of Population Health Sciences, National Health Research Institutes, 35 Keyan Road, Zhunan, Miaoli County 350, Taiwan, Republic of China; 2 Department of Nursing, Yuanpei University, No. 306, Yuanpei Street, Hsinchu 30015, Taiwan, Republic of China; 3 Department of Biostatistical Sciences, Wake Forest University School of Medicine, Medical Center Boulevard, Winston-Salem, North Carolina 27157, USA; 4 Institute of Statistics, Academia Sinica, Taiwan, Republic of China

## Abstract

The HLA region is considered to be the main genetic risk factor for rheumatoid arthritis. Previous research demonstrated that *HLA-DRB1 *alleles encoding the shared epitope are specific for disease that is characterized by antibodies to cyclic citrullinated peptides (anti-CCP). In the present study, we incorporated the shared epitope and either anti-CCP antibodies or rheumatoid factor into linkage disequilibrium mapping, to assess the association between the shared epitope or antibodies with the disease gene identified. Incorporating the covariates into the association mapping provides a mechanism 1) to evaluate gene-gene and gene-environment interactions and 2) to dissect the pathways underlying disease induction/progress in quantitative antibodies.

## Background

Rheumatoid arthritis (RA) represents a complex disease in which genes and environmental factors interplay to manifest the symptoms of this condition. Despite the phenotypic heterogeneity involved in this disease, the genetic contribution to RA is estimated to be 50-60% [1], and the HLA region has the largest influence on genetic risk. Gregersen et al. [2] showed that HLA-*DRB1 *alleles encoding a common amino-acid sequence (the shared epitope (SE)) in the third hypervariable region of the *DRB1 *molecule have been identified as risk alleles for RA. The functional significance implied by the location of this SE sequence has stimulated efforts to search for the putative RA antigen [3]. In addition, because most RA patients have autoantibody responses, including rheumatoid factor (RF, a measurement of the reactive IgM antibodies) and cyclic citrullinated peptides (anti-CCPs), Huizinga et al. [3] compared the HLA profile in a healthy population and in RA patients who did or did not produce anti-CCP antibodies, demonstrating strong interactions between the SE and anti-CCP antibodies. Based on these findings, we incorporated the number of SE alleles (categorized by NN, SN, and SS for 0, 1, and 2 SE alleles, respectively) and anti-CCP or RF into our association mapping to search for the disease locus and to assess the associations between SE, antibodies, and the disease gene in a region of 6p21 (previously identified in our linkage analyses). Two dummy variables, NN and SN, were created; SS was been treated as the reference group (NN = 1 if NN, 0 otherwise; SN = 1 if SN, 0 otherwise). Incorporating this association profile allows us to investigate associations between the disease locus and the covariates, and will help to elucidate etiopathologies between different phenotypes and the disease genes.

Recently, Liang and Chiu [4] proposed a robust multipoint association mapping approach using case-control data. This approach provides an estimate of the genetic effect and the location of the disease locus *τ*, along with sampling uncertainty to help investigators narrow down chromosomal regions that putatively harbor a disease gene (*τ*). The genetic effect denoted by "*C*" characterizes the excess disease allele frequency among cases compared to controls. Chiu et al. [5] extended this method to estimate *C *by incorporating covariates into the mapping, in order to estimate *τ *more efficiently. The covariates could be either quantitative or qualitative. Hence, we incorporated anti-CCP or IgM RF as well as the number of SE alleles into our association mapping to estimate the disease locus for RA and to assess interactions between the disease gene and the covariates simultaneously [6].

## Methods

### Materials

A total of 868 cases and 1194 controls in the North American Rheumatoid Arthritis Consortium study (NARAC) were included in analyses. We used PLINK [7,8] to clean the data, excluding two cases and one control from the data set due to their genome-wide heterozygosity <30%. We also checked the call rate, cryptic relatedness, and multidimensional scaling. No additional subjects were excluded.

From the total 35,574 single-nucleotide polymorphisms (SNPs) available originally, we excluded the SNPs with a call rate <90% or MAF (minor allele frequency) <0.05, and those that failed the Hardy-Weinberg equilibrium test (*p *< 0.05) as measured by the PLINK software. A total of 29,616 SNPs remained for further gene mapping.

The 29,616 SNPs were used to make plots for the average excess target allele frequency in cases compared to controls, divided by the estimated magnitude of linkage disequilibrium (LD) (as shown on page 146 in Chiu et al.) [5]. A region harboring 2732 SNPs with higher peaks between 28 and 40 cM was identified. A total of 1561 SNPs ranging from about 28,000 to 40,000 kb, with blocks defined by *r*^2 ^> 0.7, was selected as tag SNPs using Haploview software. We converted base pair (bp) into centimorgan (cM) approximately by dividing bp by 10^6^. Hence, our analyses focused on 706 cases with RF, anti-CCP, and number of SEs and 1191 controls with number of SEs.

### Association approaches

The method hinges on the following expression [4,5]:

including the following notations:

*D *Cases

 Controls

*t *An arbitrary location in the study region

*τ *Disease locus

: A vector of functions of covariates for cases (*s*^*D*^) and controls ()

*C *Genetic effect at *τ*

*d*_*t *_LD between t and *τ *(assuming it is independent of covariates)

 If the case carries the high-risk allele from his/her father

 If the case *does not *carry the high-risk allele from his/her father

 If the case carries the high-risk allele from his/her mother

 If the case *does not *carry the high-risk allele from his/her mother

Similarly, one can define the indicator variables,  and , for controls accordingly.

In an unmatched case-control design, all possible combinations of case-control pairs were used to localize the disease locus in this approach, a covariate for each pair being defined by a function (*g*(•)) of the case's covariate (*S*^*D*^) and the control's covariate () [for example,  or , etc.]. In this study, the covariates of antibodies were available in cases only; hence, we defined . We can show that the approach remains legitimate and the property in the Appendix in Chiu et al. [5] still holds true (unpublished data). For the covariate available in both cases and controls, , in the present study.

The genetic effect *C*, quantified by the excess high-risk allele frequency among cases compared to controls at the disease locus *τ*, can then be modeled as a function of the covariates through logistic regression [6] as follows:

The regression coefficients *β*^*T *^characterize the associations between the genetic effect at *τ *and the covariates. For covariates available for cases and controls, the approach from Chiu et al. [5] was directly applicable. On the other hand, incorporating covariates into LD mapping can improve the efficiency in estimating *τ *when the covariate carries additional information on the association under study. Because this method represents an extension of the approach proposed by Liang and Chiu [4] that is based on the generalized estimating equation approach, it is also robust, given that no assumption about the genetic mechanism is required, other than that the region contains no more than one susceptibility locus for the qualitative trait while incorporating multiple markers into the analysis simultaneously. No assumption about the underlying genetic mechanism of an incorporated quantitative covariate is required either.

The LD mapping was conducted through the sliding window approach, in which sequential analyses were performed for every 100 SNPs. We examined the associations between the estimated disease locus and the covariates in the case-control study. In addition, we compared the results from incorporating the covariates with those from the original search, where no covariates were incorporated. This allowed us to evaluate the improvement in efficiency provided by the additional information from the given covariates. To make a comparison with other association analyses, we also performed individual SNPs analysis using the chi-square test in PLINK.

## Results

**Figure 1:**
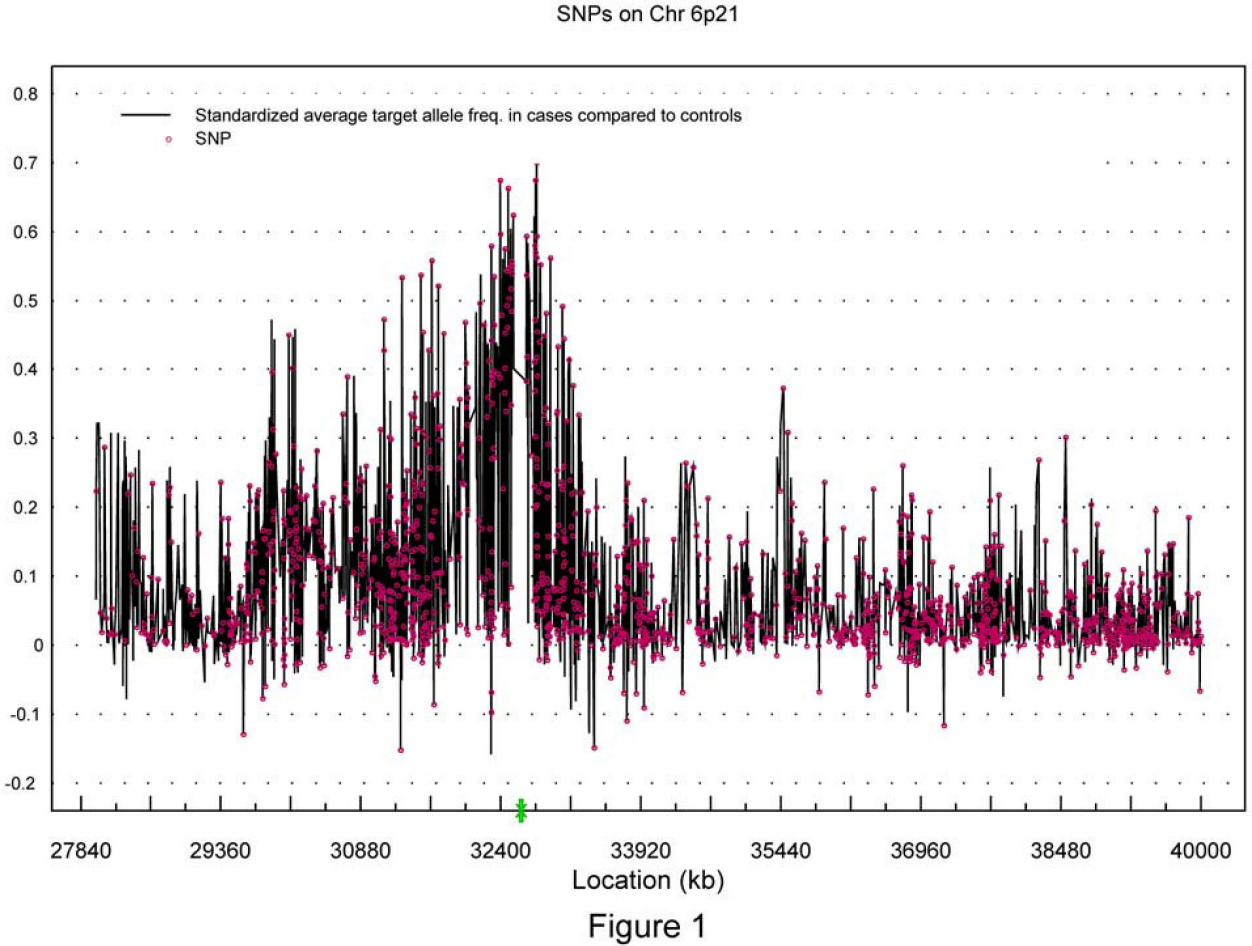
**The average excess target allele frequency in cases compared to controls divided by the estimated magnitude of LD for all SNPs located between 28,000-40,000 kp on 6p21**. Green "X", the estimated disease locus, green bracket indicates the 95% confidence interval for the disease locus.

**Figure 2:**
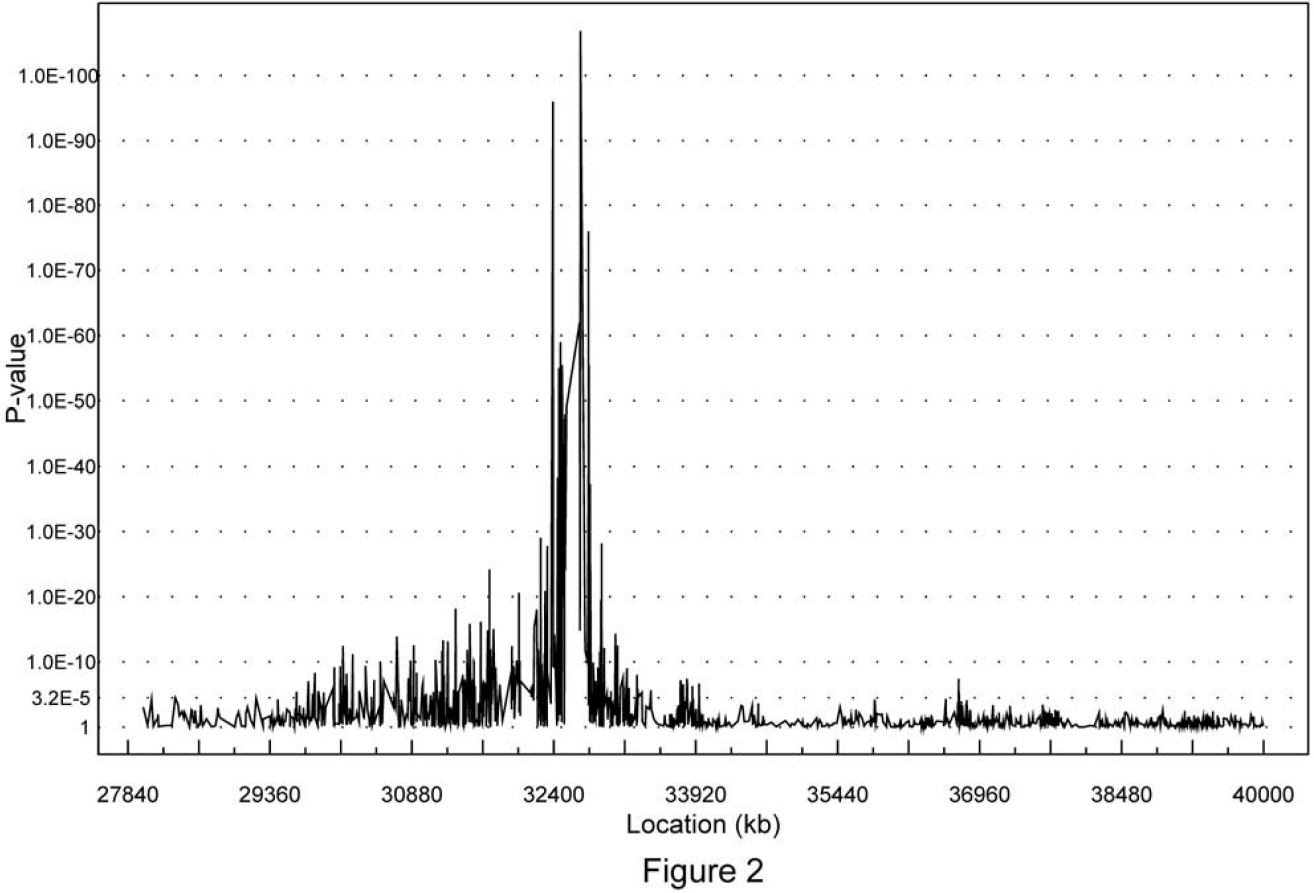
***p*-Values for individual SNPs from the association analysis based on the chi-square test**.

Figure 1 illustrates the average excess target allele frequency in cases compared to controls divided by the estimated magnitude of LD [4,5] for all SNPs located between 28,000 and 40,000 kb on 6p21. Several local peaks appeared in this region, the global peak being located within the region of 32,500-33,000 kb. Theoretically, the SNPs in high LD with *τ *or at *τ *should have a higher excess of the target allele (presumably the high-risk allele) among cases compared to controls than other SNPs in the region. Figure 1 provides an illustration of approximately where the peak (the location of *τ*) may be and whether the one-disease-locus assumption is reasonable. The results with and without incorporating covariates from the 706 cases and 1191 controls are displayed in Tables 1, 2, 3. Several local peaks were identified in this region with and without incorporating covariates (data not shown). The global peak for the estimate of *τ *was located at 32.623 cM (95% CI = [32.6191, 32.6264]): it had the highest estimated *C *value of 0.9, along with a *p*-value less than 10^-15 ^for testing *C *= 0 (the absence of association). The estimated location happened to be the locus of *HLA-DRB1 *gene, indicating that this gene is strongly associated with disease risk (i.e., it might be the disease gene or is in strong LD with the disease gene (Table 1). In the single-SNP analysis (Figure 2), 251 out of 1561 SNPs had a *p*-value ≤ 3.20 × 10^-5 ^(the significance level was chosen after applying the Bonferroni correction for multiple tests). The most significant SNP appeared to be the SNP rs660895 at 32.685360 cM (*p*-value = 1.42 × 10^-107^), as displayed in Table 4. The number of SE alleles is significantly associated with the estimated disease locus, with both *p*-values for SN and NN less than 10^-15^. There was, however, no evidence that the genetic effect at this location was associated with RF or anti-CCP levels. We also assessed the interaction between the SE numbers and the estimated disease locus by testing whether *β*_2 _(the regression coefficient for SN) = *β*_3 _(the regression coefficient for NN) (Tables 2, 3). The interactions were very significant for the estimated disease locus at 32.6 cM, revealing that these loci have interactions with SE alleles. In addition to the quantitative covariate, we also incorporated two dummy variables reflecting male-male and male-female case-control pairs, respectively, into the mapping. We also tested whether there was gene-sex interaction. The results suggested that there was no sex effect, or sex-gene interaction in our analyses (data not presented).

**Table 1:** Association mapping without a covariate based on 706 cases and 1191 controls

	*τ *(cM)	C
Estimate	32.622789	0.900
SE	0.001864	0.0480
Z		18.752
*p*-value		<1.0 × 10^-15^

**Table 2:** Association mapping with covariates RF, SN, and NN based on 706 cases and 1191 controls

	*τ *(cM)	C	*β*_1 _(RF)	*β*_2 _(SN)	*β*_3 _(NN)	Testing H_0_: *β*_2 _= *β*_3_
Estimate	32.619281	0.518	0.00790	-1.643	-2.981	
SE	0.002099		0.0301	0.0898	0.138	
Z			0.262	-18.300	-21.572	16.430
*p*-value			0.793	<1.0 × 10^-15^	<1.0 × 10^-15^	<1.0 × 10^-15^

**Table 3:** Association mapping with covariates anti-CCP, SN, and NN based on 706 cases and 1191 controls

	*τ*	C	*β*_1 _(anti-CCP)	*β*_2 _(SN)	*β*_3 _(NN)	Testing H_0_: *β*_2 _= *β*_3_
Estimate	32.618194	0.507	-0.0315	-1.533	-2.837	
S.E.	0.002109		0.0267	0.0842	0.130	
Z			-1.183	-18.217	-21.831	16.572
*p*-value			0.237	<1.0 × 10^-15^	<1.0 × 10^-15^	<1.0 × 10^-15^

**Table 4:** Association analysis of individual SNPs with a *p*-value < 1.0 × 10^-30^

SNP	Base pair	Minor^a ^allele	MAF in cases	MAF in controls	Chi-square statistic	*p*-Value	OR
rs6910071	32,390,830	G	0.5212	0.1952	435.6	1.00 × 10^-96^	4.488
rs9268368	32,441,930	G	0.6163	0.397	170.7	5.33 × 10^-39^	2.44
rs3129941	32,445,660	A	0.09517	0.2496	135.8	2.20 × 10^-31^	0.3163
rs2395157	32,456,120	G	0.5339	0.2767	247.4	9.41 × 10^-56^	2.994
rs3117098	32,466,490	G	0.1374	0.306	137	1.23 × 10^-31^	0.3612
rs2076530	32,471,790	A	0.2826	0.5553	265.5	1.08 × 10^-59^	0.3154
rs6932542	32,488,240	A	0.2144	0.4719	249.5	3.33 × 10^-56^	0.3054
rs9268615	32,510,870	A	0.5928	0.3774	165.7	6.56 × 10^-38^	2.401
rs2395173	32,512,840	A	0.1433	0.3466	185.4	3.23 × 10^-42^	0.3152
rs3129882	32,517,510	G	0.1955	0.4261	209.9	1.43 × 10^-47^	0.3273
rs7192	32,519,620	A	0.1643	0.3916	215.2	1.00 × 10^-48^	0.3055
rs6903608	32,536,260	G	0.1007	0.3116	220	8.99 × 10^-50^	0.2475
rs2516049	32,678,380	G	0.583	0.3059	279.9	8.02 × 10^-63^	3.172
**rs660895^b^**	**32,685,360**	**G**	**0.5382**	**0.1927**	**485.4**	**1.42 **× 10^-107^	**4.884**
rs5000634	32,771,540	G	0.61	0.3827	183	1.10 × 10^-41^	2.523
rs6457617	32,771,830	G	0.1861	0.491	343.6	1.03 × 10^-76^	0.2371
rs9275312	32,773,710	G	0.2953	0.1213	177.2	1.97 × 10^-40^	3.035
rs9275388	32,777,060	G	0.4942	0.2412	249.3	3.64 × 10^-56^	3.074
rs2858305	32,778,440	C	0.1551	0.3749	206.7	7.19 × 10^-47^	0.3061
rs7745656	32,788,950	A	0.1084	0.289	166.2	5.02 × 10^-38^	0.2992

^a^Minor allele based on the whole sample.

^b^Bold font indicates the most significant SNP in this region.

## Discussion

Based on Figure 1 and the analysis results, the one-disease-locus assumption seems to be reasonable for the data in this region. Because results from previous studies suggested the presence of an association between anti-CCP and SE [4], we examined the linear relationship between anti-CCP and the number of SE alleles in the 706 cases using a linear regression model. Our results suggested that the anti-CCP levels were significantly different when comparing cases with 1 and 2 SE alleles (*p *= 0.0050), but were not significantly different when comparing cases with 0 and 2 SE alleles (*p *= 0.85). Further, when we excluded the two dummy variables for SE from the association mapping, anti-CCP remained insignificant (*p *= 0.98). Similarly, excluding the two dummy variables for SE from the association mapping yielded an insignificant association with RF (*p *= 0.63). These results suggested that the association between anti-CCP or RF and the estimated disease locus was not significantly dependent on the number of SE alleles. Additionally, the estimate of the disease locus was quite consistent with the result from the association analysis of individual SNPs, based on a chi-square test. The Marshfield genetic map for this region (6p22.1-6p21.2) is 44.41-54.76 cM, spanning 10.35 cM, while by dividing by 10^6^, our map is 28 cM-44 cM, spanning 12 cM. The distances between markers will affect the results; however, because the SNP data are very dense, after converting the distances into recombination fractions, the difference resulting from two maps is likely to be very limited. The exact impact of this consideration warrants future exploration.

## Conclusion

We applied a robust multipoint LD mapping approach to locate disease genes for RA with incorporation of covariates as well as to assess whether the disease genes were associated with the covariates. Our results suggest that the disease loci in the region of 6p21 were strongly associated with the SE alleles. The efficiency in estimating the disease genes remained similar when incorporating RF or anti-CCP into the mapping, revealing that these two quantitative covariates did not provide additional information on the disease locus localization. Through this application, we demonstrated that while performing multipoint fine mapping, this approach not only facilitates examination of gene-gene interactions and gene-covariate interactions, but also helps to elucidate the pathways of complex diseases.

## List of abbreviations used

Anti-CCP: Anti-cyclic citrullinated peptide; LD: Linkage disequilibrium; MAF: Minor allele frequency; RA: Rheumatoid arthritis; RF: Rheumatoid factor; SNP: Single-nucleotide polymorphism; SE: Shared epitope.

## Competing interests

The authors declare that they have no competing interests.

## Authors' contributions

Y-FC, Y-SC, F-CH, and H-CY made contributions to the study design, statistical analysis, interpretation and draft of the manuscript. H-YK performed the data analysis.
